# Supplementary figures and images for: Genome-wide identification and characterization of non-specific lipid transfer proteins in cabbage
Source: PeerJ. 2018 Aug 10;6:e5379. doi: 10.7717/peerj.5379 (PMC6089208; doi:10.7717/peerj.5379)

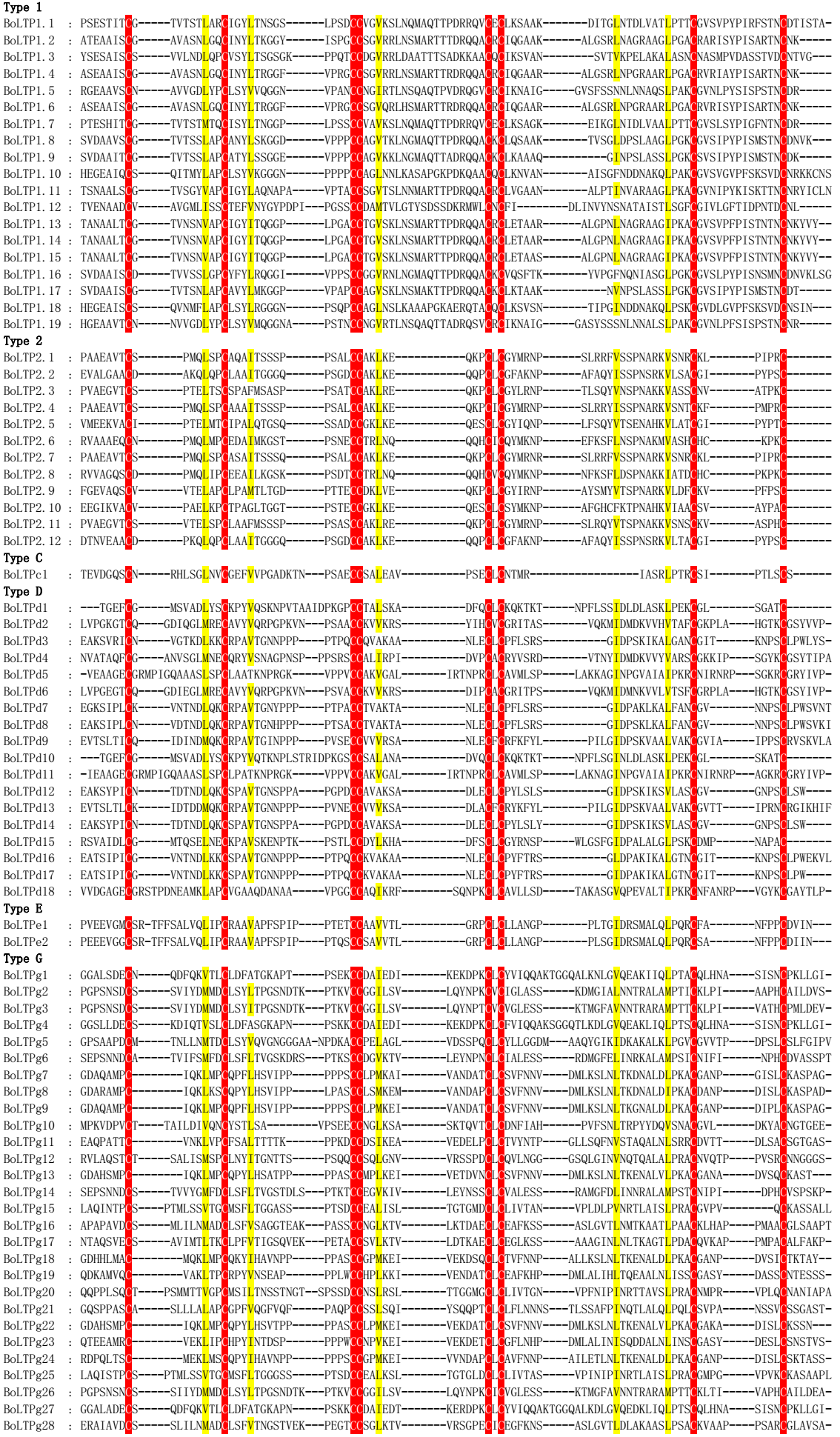

Supplement: Supplemental Information 1 — Amino acid sequences were deduced from BoLTP genes identified from the Bolbase. Sequences were aligned using MAFFT (v7.037). The conserved cysteine residues are red boxed. [file peerj-06-5379-s001.png]

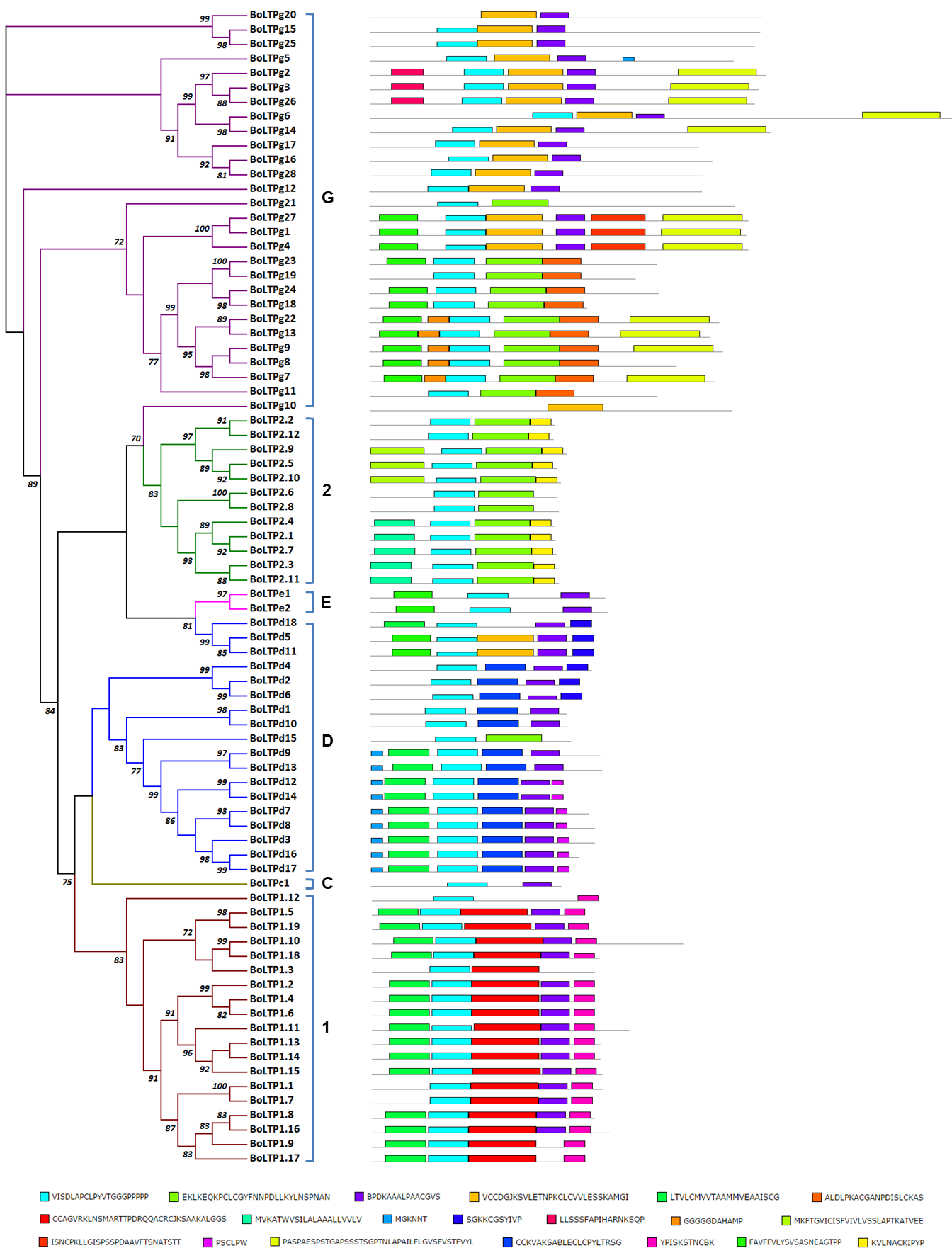

Supplement: Supplemental Information 2 [file peerj-06-5379-s002.png]

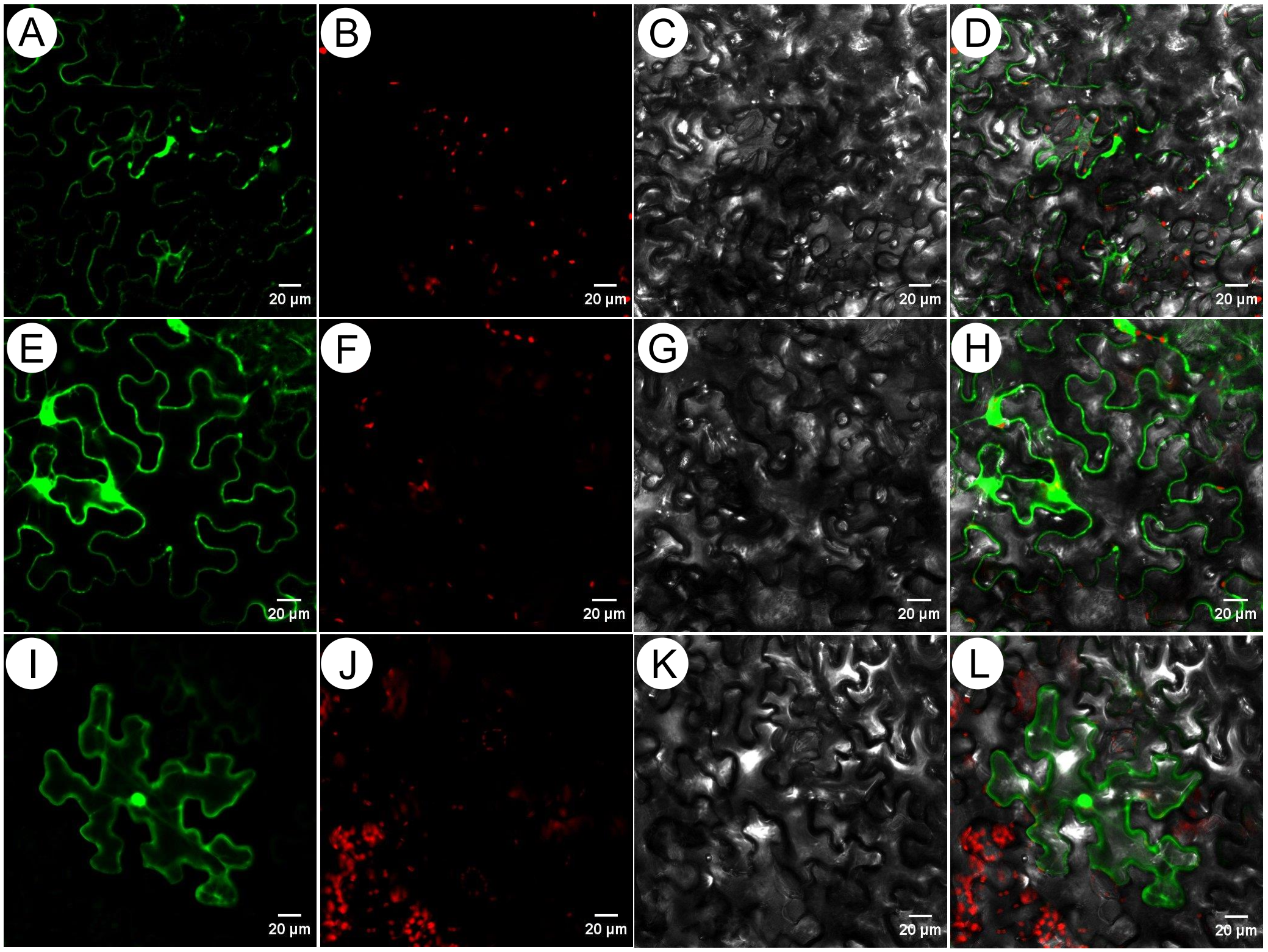

Supplement: Supplemental Information 3 — (A, E) Fluorescence signals of target genes fused with GFP. (I) Fluorescence signals of GFP alone. (B, F, J) Fluorescence signal of chloroplast. (C, G, K) Bright field images. (D, H, L) Superposition images of bright field and fluorescence. [file peerj-06-5379-s003.png]
