# Supplementary material for: Genome-wide identification and characterization of non-specific lipid transfer proteins in cabbage
Source: PeerJ. 2018 Aug 10;6:e5379. doi: 10.7717/peerj.5379 (PMC6089208; doi:10.7717/peerj.5379)
Supplement: Supplemental Information 4 — PF1 and PR1 were used for cloning of BoLTP1.7. PF2 and PR2 were used for cloning of BoLTP2.3. [file peerj-06-5379-s004.docx]

**Supplemental Table S1: Primers used for cloning of BoLTP1.7 and BoLTP2.3.** PF1 and PR1 were used for cloning of BoLTP1.7. PF2 and PR2 were used for cloning of BoLTP2.3.

| Primer name | Sequence (5’-3’) |
| --- | --- |
| PF1 | ATGGCGTTTGCTTCAAAGATCATCA |
| PR1 | CAACTACCTGTCACAATTGGTGTTG |
| PF2 | ATGGTAAAGGTGATGTGGGGTTCC |
| PR2 | TTAACATTTCGGGGTTGCAACATTGC |
