# Supplementary material for: Genome-wide identification and characterization of non-specific lipid transfer proteins in cabbage
Source: PeerJ. 2018 Aug 10;6:e5379. doi: 10.7717/peerj.5379 (PMC6089208; doi:10.7717/peerj.5379)
Supplement: Supplemental Information 5 [file peerj-06-5379-s005.docx]

**Supplemental Table S2: Cabbage genes encoding proteins with a Pfam domain PF00234 which belong to families other than the non-specific lipid transfer protein family.**

| proteins without signal peptide | Bol035192, Bol039045, Bol004245, Bol029429, Bol033989, Bol017545, Bol007675, Bol009918, Bol025594, Bol007784 |
| --- | --- |
| hybrid proline rich proteins | Bol030541, Bol029097, Bol018940, Bol000098, Bol016516, Bol018941, Bol028521, Bol045112, Bol005017, Bol029535 |
| 2S albumin storage proteins | Bol042324, Bol002555, Bol020220, Bol039712, Bol039711, Bol039713, Bol039714, Bol030540, Bol008232, Bol034483, Bol027794, Bol004580 |
