# Supplementary material for: Genome-wide identification and characterization of non-specific lipid transfer proteins in cabbage
Source: PeerJ. 2018 Aug 10;6:e5379. doi: 10.7717/peerj.5379 (PMC6089208; doi:10.7717/peerj.5379)
Supplement: Supplemental Information 6 — The EST names are GenBank accession numbers. [file peerj-06-5379-s006.docx]

**Supplemental Table S3: NsLtps used in this report, and their corresponding database accession numbers. The EST names are GenBank accession numbers.**

***M. polymorpha***

| **Name** | **EST** |
| --- | --- |
| MpLTPd1 | BJ869728 |
| MpLTPd5 | BJ855616 |
| MpLTPg3 | BJ846972 |

***P. patens***

| **Name** | **EST** |
| --- | --- |
| PpLTPd8 | BJ977311 |
| PpLTPd9 | AW561377 |
| PpLTPd11 | DC904521 |
| PpLTPd12 | DC941596 |

***S. moellendorffii***

| **Name** | **EST** |
| --- | --- |
| SmLTPd8 | FE461209 |
| SmLTPd11 | FE462950 |
| SmLTPd16 | FE457381 |
| SmLTPg6 | GT889128 |
| SmLTPg9 | FE426958 |

***A. capillus-veneris***

| **Name** | **EST** |
| --- | --- |
| AcvLTP1.1 | DK952812 |
| AcvLTP1.2 | BP917429 |
| AcvLTP1.3 | DK947840 |
| AcvLTPg2 | DK949778 |

***P. taeda***

| **Name** | **EST** |
| --- | --- |
| PtLTP1.1 | DR011430 |
| PtLTP1.3 | DN449630 |
| PtLTP1.4 | CO361240 |
| PtLTP1.6 | DN465131 |
| PtLTP2.1 | DR743561 |
| PtLTPc1 | AW982009 |
| PtLTPd4 | DR052578 |
| PtLTPd5 | DR745097 |
| PtLTPd6 | DR072724 |
| PtLTPd7 | CF403062 |
| PtLTPg1 | CF393843, DR055946 |
| PtLTPg2 | CX652807 |
| PtLTPg3 | CO363298 |
| PtLTPg4 | DR014439 |
| PtLTPg6 | CF672424 |
| PtLTPg11 | DT626206 |

***A. thaliana***

| **Name** | **Locus** |
| --- | --- |
| AtLTP1.1 | At2g15050 |
| AtLTP1.2 | At2g15325 |
| AtLTP1.3 | At2g18370 |
| AtLTP1.4 | At2g38530 |
| AtLTP1.6 | At3g08770 |
| AtLTP1.7 | At3g51590 |
| AtLTP1.8 | At3g51600 |
| AtLTP1.9 | At4g33355 |
| AtLTP1.11 | At5g59310 |
| AtLTP2.1 | At1g43665 |
| AtLTP2.2 | At1g43666 |
| AtLTP2.4 | At1g48750 |
| AtLTP2.5 | At1g66850 |
| AtLTP2.6 | At1g73780 |
| AtLTP2.9 | At3g18280 |
| AtLTP2.14 | At5g38195 |
| AtLTPc1 | At5g07230 |
| AtLTPc2 | At5g52160 |
| AtLTPc3 | At5g62080 |
| AtLTPd1 | At5g48485 |
| AtLTPd2 | At5g48490 |
| AtLTPd3 | At5g55410 |
| AtLTPd4 | At5g55450 |
| AtLTPd6 | At2g37870 |
| AtLTPd7 | At3g53980 |
| AtLTPd9 | At1g32280 |
| AtLTPd10 | At4g30880 |
| AtLTPd11 | At4g33550 |
| AtLTPe1 | At3g07450 |
| AtLTPg1 | At1g03103 |
| AtLTPg2 | At1g05450 |
| AtLTPg3 | At1g18280 |
| AtLTPg4 | At1g27950 |
| AtLTPg5 | At1g36150 |
| AtLTPg6 | At1g55260 |
| AtLTPg7 | At1g62790 |
| AtLTPg8 | At1g73550 |
| AtLTPg9 | At1g73560 |
| AtLTPg10 | At1g73890 |
| AtLTPg11 | At2g13820 |
| AtLTPg12 | At2g27130 |
| AtLTPg13 | At2g44290 |
| AtLTPg15 | At2g48130 |
| AtLTPg19 | At3g22600 |
| AtLTPg20 | At3g22620 |
| AtLTPg21 | At3g43720 |
| AtLTPg23 | At4g08670 |
| AtLTPg27 | At4g22630 |
| AtLTPg28 | At4g22666 |
| AtLTPg29 | At5g09370 |
| AtLTPg31 | At5g64080 |

***c***

| **Name** | **Locus** |
| --- | --- |
| OsLTP1.1 | Os01g12020.1 |
| OsLTP1.2 | Os01g60740 |
| OsLTP1.3 | Os03g59380.1 |
| OsLTP1.7 | Os08g03690.1 |
| OsLTP1.9 | Os11g02350.1 |
| OsLTP2.1 | Os01g49640.1 |
| OsLTP2.2 | Os01g49650.1 |
| OsLTP2.3 | Os03g02050.1 |
| OsLTP2.4 | Os05g47700.1 |
| OsLTPc1 | Os08g43290.1 |
| OsLTPc2 | Os09g35700.1 |
| OsLTPd3 | Os07g18750.1 |
| OsLTPd5 | Os01g62980.1 |
| OsLTPd6 | Os04g33920.1 |
| OsLTPd9 | Os01g58650.1 |
| OsLTPg1 | Os01g59870 |
| OsLTPg2 | Os03g07100 |
| OsLTPg3 | Os03g09230 |
| OsLTPg4 | Os03g20760 |
| OsLTPg5 | Os03g26800 |
| OsLTPg6 | Os03g26820 |
| OsLTPg8 | Os03g57980 |
| OsLTPg13 | Os06g47200 |
| OsLTPg22 | Os08g42040 |

***B. oleracea***

| **Name** | **Locus** |
| --- | --- |
| BoLTP1.1 | Bol004980 |
| BoLTP1.2 | Bol004981 |
| BoLTP1.3 | Bol007463 |
| BoLTP1.4 | Bol010613 |
| BoLTP1.5 | Bol011263 |
| BoLTP1.6 | Bol014755 |
| BoLTP1.7 | Bol014756 |
| BoLTP1.8 | Bol014771 |
| BoLTP1.9 | Bol015488 |
| BoLTP1.10 | Bol017535 |
| BoLTP1.11 | Bol017820 |
| BoLTP1.12 | Bol024824 |
| BoLTP1.13 | Bol025300 |
| BoLTP1.14 | Bol025301 |
| BoLTP1.15 | Bol025304 |
| BoLTP1.16 | Bol025938 |
| BoLTP1.17 | Bol028088 |
| BoLTP1.18 | Bol033811 |
| BoLTP1.19 | Bol033888 |
| BoLTP2.1 | Bol003276 |
| BoLTP2.2 | Bol003921 |
| BoLTP2.3 | Bol021902 |
| BoLTP2.4 | Bol022897 |
| BoLTP2.5 | Bol023407 |
| BoLTP2.6 | Bol026234 |
| BoLTP2.7 | Bol030927 |
| BoLTP2.8 | Bol039408 |
| BoLTP2.9 | Bol040181 |
| BoLTP2.10 | Bol041153 |
| BoLTP2.11 | Bol043169 |
| BoLTP2.12 | Bol045354 |
| BoLTPc1 | Bol028055 |
| BoLTPd1 | Bol003011 |
| BoLTPd2 | Bol012977 |
| BoLTPd3 | Bol015654 |
| BoLTPd4 | Bol017546 |
| BoLTPd5 | Bol017728 |
| BoLTPd6 | Bol018048 |
| BoLTPd7 | Bol018198 |
| BoLTPd8 | Bol018200 |
| BoLTPd9 | Bol018201 |
| BoLTPd10 | Bol022866 |
| BoLTPd11 | Bol025257 |
| BoLTPd12 | Bol027909 |
| BoLTPd13 | Bol027910 |
| BoLTPd14 | Bol031417 |
| BoLTPd15 | Bol033097 |
| BoLTPd16 | Bol038952 |
| BoLTPd17 | Bol038953 |
| BoLTPd18 | Bol041684 |
| BoLTPe1 | Bol005017 |
| BoLTPe2 | Bol025022 |
| BoLTPg1 | Bol002099 |
| BoLTPg2 | Bol002882 |
| BoLTPg3 | Bol005508 |
| BoLTPg4 | Bol008439 |
| BoLTPg5 | Bol009169 |
| BoLTPg6 | Bol009444 |
| BoLTPg7 | Bol009733 |
| BoLTPg8 | Bol009734 |
| BoLTPg9 | Bol009735 |
| BoLTPg10 | Bol010646 |
| BoLTPg11 | Bol010977 |
| BoLTPg12 | Bol011992 |
| BoLTPg13 | Bol013223 |
| BoLTPg14 | Bol015702 |
| BoLTPg15 | Bol018303 |
| BoLTPg16 | Bol020764 |
| BoLTPg17 | Bol024602 |
| BoLTPg18 | Bol026256 |
| BoLTPg19 | Bol026257 |
| BoLTPg20 | Bol026547 |
| BoLTPg21 | Bol030775 |
| BoLTPg22 | Bol030789 |
| BoLTPg23 | Bol039422 |
| BoLTPg24 | Bol040003 |
| BoLTPg25 | Bol040990 |
| BoLTPg26 | Bol041658 |
| BoLTPg27 | Bol041803 |
| BoLTPg28 | Bol042588 |
| BoLTPx1 | Bol010647 |
| BoLTPx2 | Bol014710 |
| BoLTPx3 | Bol019670 |
| BoLTPx4 | Bol021025 |
| BoLTPx5 | Bol021026 |
| BoLTPx6 | Bol033092 |
| BoLTPx7 | Bol043398 |
| BoLTPx8 | Bol043752 |
| BoLTPx9 | Bol044131 |
